# Procrastination in University Students: A Proposal of a Theoretical Model

**DOI:** 10.3390/bs13020128

**Published:** 2023-02-02

**Authors:** Luis Araya-Castillo, Mildred Burgos, Patricia González, Yuracid Rivera, Nicolás Barrientos, Víctor Yáñez Jara, Francisco Ganga-Contreras, Walter Sáez

**Affiliations:** 1 Facultad de Ingeniería y Empresa, Universidad Católica Silva Henríquez, Santiago 8330225, Chile; 2 Escuela de Administración y Negocios, Universidad Miguel de Cervantes, Santiago 8320170, Chile; 3 Escuela de Psicología, Universidad Miguel de Cervantes, Santiago 8320170, Chile; 4 Facultad de Administración y Negocios, Universidad Autónoma de Chile, Providencia 7500912, Chile; 5 Instituto de Salud Pública, Universidad Andrés Bello, Santiago 7591538, Chile; 6 Facultad de Educación y Humanidades, Universidad de Tarapacá, Arica 1000007, Chile

**Keywords:** procrastination, theoretical model, higher education

## Abstract

Procrastination is a phenomenon that affects university students and consists of not finishing a task or finishing it late, which has a direct impact on their academic performance. This is relevant because, in a context of high competition, higher education institutions and their decision-makers need to be aware of the factors that influence university students’ procrastination in order to implement actions that favor student attraction and retention. Based on the above, this research aims to propose a theoretical model of procrastination in university students, based on the literature review and content validation assessment through a semi-structured questionnaire. The proposed model is made up of nine dimensions: Psychological, Physiological, Social, Academic, Leisure, Time Management, Resources, Labor, and Environmental. Dimensions were obtained based on adequate levels of content validity provided by the literature and the questionnaire. In the future, the research proposes to study the way in which these dimensions are present in procrastination and design a scale that allows for their measurement.

## 1. Introduction

In our daily lives, we have to perform multiple tasks in different areas. This leads people to two paths: carrying out the task as soon as possible or postponing it; the latter being part of the tendency to delay the start or completion of a task [1], an act also known as procrastination [2]. This situation is not isolated: some research studies have found that the majority of people who procrastinate are young adults, [3,4,5]. Studies conducted in Latin America concluded that approximately 61% of people show some level of procrastination, while 20% do so on a regular basis [6,7]. This behavior is therefore present in a large majority of people, affecting areas of life such as work, health, and academia [8]. However, the fact that most affected people are young raises questions about the influence of procrastination in academia and higher education, a system that is relevant to every society. So much so that some people believe that the wealth or poverty of countries depends to a large extent on the quality of their higher education [9] as it is considered a key element in economic prosperity [10], vital for social progress [11], the central link in developing talent and culture [12], and essential for sustainable development and improvement of people’s well-being [13].

The above serves as a context for the purpose of this study, which is to propose a theoretical model of the factors involved in academic procrastination in university students. This is relevant because education basically focuses on the progressive development of students’ knowledge and skills, and on the creation of an environment of safety and healthy interaction among students, professors, and the rest of the people in the institution [14,15,16]. Understanding the factors that explain procrastinating behavior is not only relevant for students and universities but also for society as a whole, since, among all sectors, higher education is the one that relates the most to the growth of a society and its socio-economic development [17].

Therefore, this research studies the higher education sector in a way that is rigorous, practical, and functional for public policymakers and managers [18]. This analysis does not only have implications for Chile, as it shows that education has similar dynamics in other countries, except perhaps in low-income countries [19]. In order to meet the study aim, a literature review on academic procrastination was carried out based on a multidimensional approach, reviewing theories and areas of application in Latin American countries since 2008. As a result, six major dimensions were identified that cover the related aspects. Results were transferred to a semi-structured questionnaire for content validation and then applied to a theoretical sample. The responses identified nine dimensions, of which four are repeated from the theory (psychological, academic, physiological, and environmental dimensions) and five are highlighted from the questionnaire (social, time management, resources, leisure, and labor dimensions).

### 1.1. Procrastination

Procrastination is a relatively old phenomenon, as psychologist William James already recognized the emotional cost generated in people who suffered from it more than 120 years ago [20]. Lay [21] pointed out that procrastination considers importance to the individual whose action is being postponed, while Milgram, Mey-Tal, and Levison [22] discussed whether the performance of a task is voluntary or imposed, and Steel [2] wondered whether the person is aware of the negative consequences of this postponement [23].

Along these lines, Steel and Ferrari [5] defined procrastination as an insufficiency in self-regulation processes that causes the voluntary delay of planned activities [24] or an ineffective lifestyle that leads to a failure in the fulfillment and commitment to set targets. In this light, procrastination involves the action of not finishing a task or finishing it late, and this process is generally accompanied by feelings of nervousness or restlessness, and discouragement [25].

This habit is considered destructive since the cost of such behavior may cause psychological stress due to overexertion to meet deadlines [26], when not fulfilling responsibilities may lead to negative consequences and when the positive consequences of performing the task are higher [27,28].

The literature on the topic has been intensified in recent years, with several descriptive studies relating procrastination to other variables [24], including its status during the COVID-19 pandemic [29].

### 1.2. Procrastination in the Academic Field

In the academic field, procrastination can be defined as a behavior that involves always or almost always postponing the start or completion of academic tasks, or always or almost always experiencing problematic levels of anxiety associated with such postponement [30]. Likewise, Álvarez-Blas [31] defined academic procrastination as an unnecessary and unjustified delay in study-related tasks. In line with this, Schouwenburg [32] pointed out that there are two types of academic procrastination: sporadic and chronic. Sporadic academic procrastination refers to a one-off behavior while chronic academic procrastination is the generalized habit of delaying studying [33].

Literature relates procrastination to other (moderating or controlling) variables, such as self-efficacy [34], fear of failure [35] task aversion [36], lack of self-regulation [37], or disruptive classroom behavior [38], among others. Likewise, different studies have found that academic procrastination is associated with factors generated by academic tasks and limited time planning skills [39,40,41,42,43,44].

Other research studies aimed at understanding and explaining the manifestations of procrastination in everyday and academic life have associated them with mental representations; this may be because students may be imagining something that is not explicit or observable in the environment, but that is a problem that they somehow need to solve [45]. In line with this argument, a study conducted in Ecuador links performance and emotional regulation as predictors of academic procrastination in university students, with no difference between men and women [46].

Other authors have also shown a negative correlation between academic performance and university procrastination [47,48,49]. Although an ideal university student is successful, the demands of higher education lead to a high percentage of students not being able to achieve academic success due to procrastinating behavior among other issues [50].

In line with this, Cardona [51] mentioned that academic procrastination entails various consequences, including poor academic performance, demotivation, burnout, academic stress, and even dropping out of school. This is relevant as an estimated 80% to 90% of university students show dilatory behaviors at some point [24].

It is therefore possible to argue that there are multiple factors associated with dropping out and/or academic failure in higher education, and the emphasis on a specific factor depends on the authors’ perspective [52,53,54,55,56,57,58,59,60].

The postponement of responsibilities is a phenomenon characteristic of modern societies. Academic procrastination is a variable of interest in the context of higher education due to the consequences of homework avoidance at the university. Academic procrastination has been studied by different disciplines or explanatory models, mainly in the field of psychology. However, since this is a phenomenon of human behavior, it is to be assumed that there are other factors that intervene in this condition.

Rosario et al. [61] conducted research on this variable in non-Latin American contexts and found out that about 20% of adults admit to procrastinating in routine tasks, about 25% of the adult non-student population report that procrastination is a significant problem, and in 40% of cases it has caused financial loss at both personal and organizational levels.

As for academic procrastination, studies by Tice and Baumeister [62] and Landry [63] have proven the extent of the problem, where approximately 20% of United States college students show persistent academic procrastination, while behaviors associated with procrastination are observed in more than 80% of them. Additionally, studies such as those by Rothblum, Solomon, and Murakami [64], Rothblum [65] and Tice and Baumeister [62] show that academic procrastination is significantly related to poor academic performance, personal stress, and physical health in college students [63]. These authors identify influential factors such as performance, personal stress, and health.

While there is a great deal of research on academic procrastination in Latin America, there is little research on the specific elements associated with the concept. Therefore, it is necessary to find reliable information and data to determine the variables that influence academic procrastination in detail, considering the students’ environment, cultural context, and socio-demographic variables, among others. This also explains the interest in the theoretical review of the variable in order to find the elements that influence the concept.

## 2. Materials and Methods

**Figure 1:**
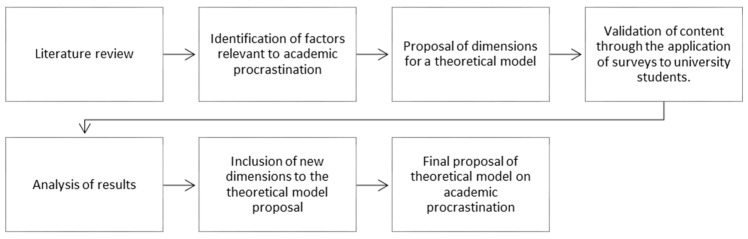
Summary of research stages.

This research is of a conclusive descriptive nature and a cross-sectional or sectional cut [66]. The aim of this study is to suggest a theoretical model for university procrastination based on background information from the literature, and validate it by applying a semi-structured questionnaire to a theoretical sample. To search for dimensions of the theoretical model of procrastination in university students, a literature review was carried out on different scales applied to this topic in the Scopus and Scielo databases, using the concepts (in English and Spanish) of “procrastination scales”, “academic procrastination”, “university procrastination”, and “student procrastination”, and focusing on the research in Latin American countries between 2008 and 2022. Scopus is used because it is the multidisciplinary database with the largest number of journals, in addition to having a strong Latin American presence in its catalog [67]. Scielo, on the other hand, is used because it is a regional database that integrates geographic collections from Latin American and African countries, whose main purpose is to make science generated in these territories visible [68,69]. The literature review made it possible to identify a series of factors that were grouped into six dimensions proposed by the researchers, determined mainly by their closeness or linkage to the concept according to the research reviewed. The results of this search were used later in an online questionnaire (conducted on the SurveyMonkey platform) completed mainly by students belonging to a group of Chilean universities located in the metropolitan region who volunteered to do so. The survey was applied from August 2017 to August 2019, and its dissemination was carried out through different academic networks that the authors have in the universities of the metropolitan region. A total of 320 responses were obtained from those invited to participate. All participating subjects gave their informed consent at the time of answering the questionnaire. The sample was non-probabilistic by convenience and consisted of students from different universities, programs, and modes of study. In the questionnaire, respondents were asked to write down the factors that best represent procrastination in academic tasks. This information allowed us to identify the dimensions and items that should be considered in the model proposal. Content validity was understood as the degree to which the measure captures the domain of the concept under study [70]. Content validity is relevant in the development stage of the measurement instrument as it shows that the indicators included in the survey are a representative sample of the set to be used [71]. Figure 1 summarizes the above steps.

### Sample Description

The sample consisted of 320 people living in Chile, 62.19% of whom are female and 37.81% are male. The age ranges from 18 to 62 years and most respondents (79.69%) live in the metropolitan region, 8.13% in Valparaíso, 4.69% in the Bío-Bío region, 3.13% in the Araucanía region, 0.63% in the Tarapacá region, and 0.31% in the Coquimbo region. Respondents were also asked to specifically report their municipality of residence. The most frequently reported municipalities are Santiago, Puente Alto, La Florida, Ñuñoa, Maipú, Cerrillos, Quilicura, Lampa, Paine, Pudahuel, Renca, Maule, etc. As for their nationality, 96.88% are Chilean, while 3.12% are foreign nationals. The level of education of the sample was as follows: 63.75% of respondents have not completed a university undergraduate degree, 9.69% have completed a university undergraduate degree, 7.50% have completed secondary education, 5.63% have not completed a university postgraduate degree, 3.75% have completed a higher level technical degree, 3.44% have not completed a higher level technical degree, 2.50% have not completed studies at a professional institute, 2.19% have completed studies at a professional institute, and 1.55% have completed a university postgraduate degree. As for the level of undergraduate study, the most frequently reported degrees by respondents were audit, commercial engineering, psychology, human resources, social work, and accounting. As for employment, 44.69% of respondents are students, 40% study and work, 8.75% work, 4.06% are unemployed job seekers, and 2.50% are not in paid employment. As for the level of household income, 18% have an income between USD 0 and 336 (CLP 0 and 300,000), 29.93% have an income between USD 336 and 672 (CLP 300,000 and 600,000), 20.44% have an income between USD 672 and 1008 (CLP 600,000 and 900,000), 14.75% have an income between USD 1008 and 1344 (CLP 900,000 and 1,200,000), and 16.88% have an income over USD 1680 (CLP 1,500,000). In terms of the composition of the family unit, respondents report a range from two to five members.

## 3. Results

### 3.1. Literature Review

Based on the literature review and the search of a multidimensional approach to the variable, theoretical advances and areas of application of academic procrastination were reviewed. The aim was to carry out a complete review of the literature regarding studies that suggest and/or validate models (scales) of university procrastination and their dimensions in Latin-American countries. This is shown in Table 1.

Following the literature review, a total of 37 papers were found that use models with scales. This is the background for proposing the theoretical model: “Procrastination of University Students” (PUS). The PUS model is multidimensional and reflexive, as the latent variable (procrastination construct in university students) causes the observed variables (procrastination dimensions) [102]. The first proposal of the theoretical model is composed of 6 dimensions linked to procrastination in higher education students; namely: (1) Psychological; (2) Physiological; (3) Socio-demographic; (4) Academic; (5) Cultural; and (6) Environmental. The theoretical model considers the dimensions that are mostly incorporated by the studies in the academic literature, as well as the aspects or elements considered by each study. Table 2 shows detailed information on the dimensions, considered aspects, and authors.

The six proposed dimensions cover all the relevant aspects reviewed in the literature; therefore, they can be used for content validation. The results show that the “Psychological” dimension includes factors such as motivation, personality dimensions, emotional intelligence, time management, and so on. The “Physiological” dimension is composed of biological variables: anxiety, self-regulation of learning, and vigor, among others. The “Socio-demographic” dimension is made up of aspects, such as gender, age, socio-economic and demographic variables. The “Academic” dimension includes control variables, distance learning, academic performance, level of education, dropping out, academic performance, GPA, study habits, and academic satisfaction, among others. The “Cultural” dimension mainly contains personal individual elements. Lastly, the “Environmental” dimension involves aspects such as risk factors, difficulties in performing tasks, lack of time, levels of dedication, and peer influence.

### 3.2. Questionnaire

The literature review yielded six dimensions that theoretically link a number of elements with procrastination in university students. These dimensions were assessed through a semi-structured online questionnaire on a sample of 320 respondents studying in Chilean universities. Respondents were asked to write down the factors or dimensions that best represent procrastination in academic tasks and relate them to a number of items on the topic.

As background information, respondents were asked several questions about how they deal with tasks on a daily basis from the point of view of procrastination. Table 3 shows the results when the respondents were asked to choose an item with which they most identify. From a total of 309 responses, 60.95% of respondents identified most with “I do my tasks in order of priority”; 19.37% with “I do my tasks whenever I have time”; 16.51% with “I do my tasks depending on the backlog”; 1.9% with “I do not do any task”; and 1.27% with “I only do the tasks I manage to get done”.

In order to validate the content of the first proposal of the Theoretical Model “Procrastination of University Students”, the following question was asked: “What are the dimensions that best represent procrastination in academic tasks?” A total of 320 responses were collected, covering nine dimensions, from which four are repeated in the literature review and five enhanced their importance thanks to the questionnaire. The dimensions are Psychological, Social, Academic, Physiological, Time Management, Leisure, Resources, Environmental, and Labor. Table 4 shows the importance given by respondents to each dimension and its component elements.

The dimension that best represents procrastination in academic tasks according to the respondents was the Psychological one, with 22.91% of preferences, and the “lack of motivation” factor was the most chosen one (22.24%). This was followed by the Social dimension, with 19.34% of preferences, with the factor “family aspects” as the most relevant with 28.83%. In third place was the Academic dimension with 13.15%, where the “educational model” was the most representative factor with 21.85% of preferences. The Physiological dimension reported 12.28% of preferences, where “physiological needs” was the most relevant factor (26.6%). Time Management ranked fifth among the dimensions, with 9.58%, and “lack of time” as the dominant factor with 40.91%. The Leisure dimension emerged as a new dimension with 9.28% of preferences, with “recreational activities” as the most chosen factor (30.03%). Resources and Environmental dimensions showed similar percentages, with 5.31% and 5.10%, respectively, where “economic resources” dominated the former (84.43%), and “conflictive environment” the latter (41.03%). The Labor dimension least represented procrastination according to the respondents, with “workload” as the dominant factor with 70% of preferences.

### 3.3. Limitations

Owlia and Aspinwall [103] established that validity comprises two aspects: quantitative validity and qualitative validity. Qualitative validity identifies the dimensions linked to procrastination in the literature and reviews their validity through a questionnaire. Qualitative validity determines whether the measures capture the key factors of an unobservable construct, which is also in line with content validity. The latter is relevant for full validation and the subsequent scaling of the PUS model. Future research studies are expected to collect information through individual interviews, focus groups, and expert opinions, seeking to achieve category saturation [104]. The proposal, therefore, is to use qualitative tools as they allow for analyzing phenomena in greater depth than quantitative tools [105]. The proposal includes the following: fifteen in-depth interviews to be conducted to find out students’ perceptions of the procrastination construct and the dimensions that they consider relevant in evaluation; four focus groups to study and analyze students’ perceptions in interaction; and four experts in university procrastination can also participate, who should be asked to evaluate the proposed scale for procrastination in university students. This information will be used to validate the results obtained from the semi-structured online questionnaires applied to the students.

## 4. Conclusions

This research studies the factors that influence procrastination among university students in Latin America on this topic because none of the previous studies have covered the factors affecting this concept. The aim was to collect information in relation to the actions that have an impact on the backlog of academic tasks. After data collection, the dimensions that directly affect the student’s procrastination were considered through a repetitive search, and the content validity was given by the questionnaire. The analysis seems to conclude that the factors that mainly influence university students to postpone the fulfillment of an obligation or the development of action have to do with the psychological, social, and academic dimensions and the factors that integrate them.

These results are in line with other international studies that deem these dimensions relevant. Even so, they also highlight areas such as resources and labor, which for a certain percentage of students are distracting elements that prevent the fulfillment of their academic tasks. This is not a novelty, since due to the configuration of higher education systems in Latin America (and in Chile), many students must work in order to pay for their university studies, making both roles compatible. Academic procrastination is not a subject of the establishment of a repertoire of activities but is rather a self-regulatory model that includes aspects such as autonomous learning goals.

The aim of the research was to propose a theoretical model of procrastination in university students, which was achieved by means of quantitative instruments. However, the model still needs to corroborate its full validation through qualitative methods in order to carry out the corresponding factorial analysis, which will make the model reliable and accurately prove that procrastination in university students is explained by the aforementioned dimensions. This is not a trivial task, as a scale measuring procrastination in university students can finally be developed.

## Figures and Tables

**Table 1:** Previous Scales on Procrastination.

Author(s)	Country of Application	Scope of Application	Dimensions	Factors
Pichen-Fernández and Turpo Chaparro [72]	Peru	University students	2	Procrastination, self-efficacy
Altamirano and Rodríguez [73]	Ecuador	University students	3	Procrastination, anxiety, gender
Aspée and Herrera [74]	Chile	University students	2	Procrastination, student engagement
Estremadoiro, Parada, and Schulmeyer [8]	Bolivia	University students	4	Procrastination, time management, psychological processes, academic performance
Alegre-Bravo and Benavente-Dongo [75]	Peru	University students	2	Procrastination, academic performance
Burgos-Tower and Salas-Bla [76]	Peru	University students	2	Procrastination, self-efficacy
Silva, Machado, Lays, and Fonsêca [77]	Brazil	University students	2	Motivation, socio-demographic variables
Mejía, Ruiz-Urbina, Benites-Gamboa, and Pereda-Castro [78]	Peru	University students	3	Procrastination, gender, age, academic variables
Andrade-Gámez [79]	Ecuador	High school students	3	Gender, procrastination, motivation
Domínguez-Lara and Campos-Uscanga [80]	Peru	Psychology students	2	Procrastination, satisfaction with studies
Garzón-Umerenkova and Gil-Flores [81]	Spain	University students	4	Time management, dropout, distribution of time, academic performance
Rodríguez and Clariana [24]	Spain	University students	3	Age, procrastination, academic year
Garzón-Umerenkova and Gil-Flores [82]	Colombia	Higher Education	7	Attraction to the task, love of the task, uncertainties about the task, fear of failure, task failure, perfectionism, academic achievement
Marquina-Luján, Gómez-Vargas, Salas-Herrera, Santibañez-Gihua, and Rumiche-Prieto [83]	Peru	University students	2	Procrastination, socio-demographic profile
Navarro-Roldán [84]	Colombia	Psychology students	3	Procrastination, motivation, control variables
Pardo-Bolívar, Perilla-Ballesteros, and Salinas-Ramírez [85]	Colombia	Higher Education	4	Boredom, time management, peer influence, and health hazards
Tarazona-Pérez, Romero-Acuña, Aliaga-Contreras, and Veliz-Rodríguez [86]	Peru	Higher Education	3	Indecision, avoidance, activation
Yarlequé, Alva, Nuñez, Navarro, Padilla, Matalinares, Navarro, and Cárdenas [87]	Peru	University students	3	Stress, procrastination, psychological well-being
Medina and Tejada [88]	Peru	University students	2	Procrastination, self-esteem
Rico-Palma [89]	Chile	University students	4	Procrastination, age, gender, motivation
Sureda-Negre, Comas-Forgas and Oliver-Trobat [90]	Spain	Higher Education	3	Time, teaching model, assessment
Angarita-Becerra [91]	Argentina	Human behavior	3	Psychological processes, sources of motivation, behavior
Domínguez-Lara, Villegas-García and Centeno-Leyva [92]	Peru	Higher Education	5	Grade Point Average (GPA), study habits, academic satisfaction, expectation to succeed, motivation
Furlan, Ferrero, and Gallart [93]	Argentina	University students	5	Anxiety, mental symptoms, procrastination, academic performance, gender
Paz, Aranda, Juana, Delgado, and Sayas [45]	Peru	University students	4	Risk factors, protective factors, lack of time, stress
Segovia [94]	Uruguay	University students	3	Gender, age, socio-economic status
Carranza and Ramírez [4]	Peru	University students	2	Gender, age
Clariana [95]	Spain	University students	3	Procrastination, personality dimensions, dishonest behavior
Furlan [96]	Argentina	Higher Education	3	Anxiety, academic self-efficacy, self-regulation of learning
Guzmán-Pérez [23]	Spain	University students and human behavior	4	Procrastination, anxiety, demographic variables, biological variables
González-Brignardello and Sánchez-Elvira [97]	Spain	Engagement	3	Dedication, vigor and absorption, commitment
Clariana, Cladellas, Badía, and Gotzens [43]	Spain	Psychology students	5	Procrastination, academic delay, emotional intelligence, gender, age
Galarregui, Arana, and Partarrieu [98]	Argentina	Academia	2	Procrastination, perfectionist traits
Sampaio and Bariani [99]	Brazil	Higher Education	4	Lack of time, dissatisfaction in performing tasks, not having time, difficulty performing tasks
Alvarez-Blas [31]	Peru	High school students	3	Procrastination, gender, level of education
Böhrt, Arce, Walker, and Romero [100]	Bolivia	University students	3	Procrastination, distance learning, perfectionism
Rosario, Núñez, Salgado, González-Pienda, Valle, Joly, and Bernardo [101]	Spain	High school students	3	Procrastination, personal variables, anxiety

**Table 2:** Proposal for a Theoretical Model of Procrastination in University Students.

Dimensions	Considered Aspects	Previous Scales
Psychological	Motivation, personality dimensions, dishonest behavior, perfectionism, self-esteem, psychological well-being, emotional intelligence, perfectionist traits, time management, time distribution, mental symptoms, psychological processes, dissatisfaction with performing tasks, self-regulation, expectation to succeed, indecision, avoidance, activation, self-regulation, sources of motivation, behavior, postponement of the task, commitment, attraction to the task, fear of failure, boredom, uncertainty about the task.	[8,23,43,73,74,77,79,81,82,84,85,86,87,88,89,91,92,93,95,97,98,99,101]
Physiological	Biological variables, anxiety, stress, self-regulation of learning, vigor, absorption, health hazards.	[23,45,85,87,93,96,97,101]
Socio-demographic	Gender, age, personal variables, socio-demographic profile, socio-economic level, demographic variables.	[4,23,24,31,43,77,78,79,83,89,93,94]
Academic	Dropping out, control variables, distance learning, academic performance, academic year, level of education, academic performance, GPA, study habits, academic satisfaction, study patterns, exam performance, academic delay, academic self-efficacy, teacher model, assessment, academic behavior.	[8,24,31,43,72,75,76,78,80,81,82,84,87,90,92,93,96,100]
Cultural	Personal variables, planning failure.	[101]
Environmental	Risk factors, protective factors, difficulty in performing tasks, lack of time, time, dedication, peer influence.	[45,85,90,97,99]

**Table 3:** Respondents’ Results on How they Deal with Tasks.

Items	Responses
I do my tasks in order of priority	188
I do my tasks whenever I have time	59
I do my tasks depending on the backlog	52
I do not do any task	6
I only do the tasks I manage to get done	4

**Table 4:** Percentage of importance for each dimension and its associated factors.

Dimension	Preference Percentage	Associated Factors
Psychological	22.91%	Lack of motivation, attitude, insecurity in personal skills, psychological factors, stress, boredom, exciting factors, and addictions
Social	19.34%	Family aspects, socialization, socio-cultural level, personal problems, lack of support, home activities, and love relationships
Academic	13.15%	Educational model, teacher performance, study habits, administrative-academic management of the university, academic training, interest in the task, academic overload, vocation, and university environment
Physiological	12.28%	Physiological needs, physical or mental exhaustion, depression, health status, age, pregnancy, Attention Deficit Disorder (ADD)
Time Management	9.58%	Lack of time, definition of priorities, task planning, doing everything at the last minute and task overload
Leisure	9.28%	Recreational activities, technological devices, social media, browsing the Internet, and leisure time
Resources	5.31%	Transportation, economic resources, academic resources, and technological resources
Environment	5.10%	Conflicting environment, accessibility, environmental conditions, inappropriate study space and distracting elements
Labor	3.05%	Workload, working and studying at the same time, work problems, and working hours.
